# Inhibitory Control in Bulimic-Type Eating Disorders: A Systematic Review and Meta-Analysis

**DOI:** 10.1371/journal.pone.0083412

**Published:** 2013-12-31

**Authors:** Mudan Wu, Mechthild Hartmann, Mandy Skunde, Wolfgang Herzog, Hans-Christoph Friederich

**Affiliations:** Department of General Internal Medicine and Psychosomatics, University of Heidelberg, Heidelberg, Germany; University of Western Brittany, France

## Abstract

The aim of this meta-analysis was to summarise data from neuropsychological studies on inhibitory control to general and disease-salient (i.e., food/eating, body/shape) stimuli in bulimic-type eating disorders (EDs). A systematic literature search was conducted to identify eligible experimental studies. The outcome measures studied included the performance on established inhibitory control tasks in bulimic-type EDs. Effect sizes (Hedges' *g*) were pooled using random-effects models. For inhibitory control to general stimuli, 24 studies were included with a total of 563 bulimic-type ED patients: 439 had bulimia nervosa (BN), 42 had anorexia nervosa of the binge/purge subtype (AN-b), and 82 had binge eating disorder (BED). With respect to inhibitory control to disease-salient stimuli, 12 studies were included, representing a total of 218 BN patients. A meta-analysis of these studies showed decreased inhibitory control to general stimuli in bulimic-type EDs (*g* = −0.32). Subgroup analysis revealed impairments with a large effect in the AN-b group (*g* = −0.91), impairments with a small effect in the BN group (*g* = −0.26), and a non-significant effect in the BED group (*g* = −0.16). Greater impairments in inhibitory control were observed in BN patients when confronted with disease-salient stimuli (food/eating: *g* = −0.67; body/shape: *g* = −0.61). In conclusion, bulimic-type EDs showed impairments in inhibitory control to general stimuli with a small effect size. There was a significantly larger impairment in inhibitory control to disease salient stimuli observed in BN patients, constituting a medium effect size.

## Introduction

The spectrum of bulimic-type eating disorders (EDs) includes anorexia nervosa of the binge/purge subtype (AN-b), bulimia nervosa (BN), and binge eating disorder (BED). These conditions share the common characteristic of recurrent episodes of binge eating (i.e., overeating of large amounts of food) with (AN-b and BN) or without (BED) compensatory behaviours, e.g., self-induced vomiting. Binge eating is associated with a ‘definite sense of loss of control’ [1]. Although the bulimic-type EDs are mental disorders associated with increased morbidity and all-cause mortality [2], [3], the mechanisms underlying bulimic behaviours are largely unknown.

In addition to the categorical classification of EDs, in recent years and with the upcoming fifth edition of the Diagnostic and Statistical Manual of Mental Disorders (DSM-5), a renewed interest in dimensional concepts and trans-diagnostic theories in EDs (AN, BN, BED) can be observed [4]–[6]. Recurrent episodes of binge eating are considered to be a common phenomenon in purging spectrum disorders and are evident in various ED pathologies, including AN (binge/purge subtype), BN and BED. Furthermore, impaired inhibitory control in bulimic-type ED patients does not appear to be restricted to food intake but may extend to general behaviours, e.g., excessive drinking, substance abuse, sexual disinhibition, and bullying, suggesting a more general dysregulation of inhibitory control in bulimic-type EDs [7], [8].

Neurocognitive tasks have been developed to investigate inhibitory control in and across different psychopathologies. However, there is a lack of systematic evidence from neuropsychological studies assessing and comparing inhibitory control capacities across different ED diagnoses. Because studies using subjective ratings of impulse control capacities and neuropsychological data generally fail to show strong associations (e.g., [9]–[11]), the assessment of inhibitory control with more objective and well-defined behavioural and cognitive tasks is of great importance.

Inhibition is a broad term that describes a wealth of phenomena. The present review is concerned with voluntary inhibition, as a subcomponent of cognitive control functions. Inhibitory control refers to the ability to suppress, interrupt, or delay an activated behaviour or cognitive course of action [12]–[15]. Inhibitory control is not a unitary construct, but consists of several subcomponents. These share a common underlying neural network but the degree of regional involvement seem to differ between the subcomponents [16]. A more basic distinction may exist between inhibitory mechanisms that control overt behaviors (i.e., behavioural inhibition) and those that control mental and attentional processes (i.e. cognitive inhibition). Based on the review of Bari et al. [16], the concept of behavioural inhibition encompasses the subdivision of response inhibition, reversal learning and delayed gratification. In the present meta-analysis, we limit our analysis to response inhibition and disinhibition, as reversal learning and delayed gratification depend on several additional factors (i.e. set-shifting, learning, reward sensitivity, decision making) for successful performance. Furthermore, compulsivity and reversal learning have been addressed in previous meta-analysis of ED patients (e.g., [17]). The concept of cognitive inhibition instead encompasses cognitive and attentional processes such as when during attentional processing task-irrelevant information has to be suppressed (i.e., interference control) [13].

Response inhibition tasks have been most commonly used in ED studies to investigate inhibitory control (behavioral inhibition). The most prominent examples are the No-go task [18] and the Stop Signal Task [12], which measures an overt, effortful expression of inhibitory control involving the suppression of an activated behavioural response [13]. In the classical No-go task, participants must respond to a frequently presented target and to inhibit their responses to an infrequently presented non-target [19]. The SST differs from the No-go task in that participants must inhibit an already initiated motor response [12]. The number of inhibitory control failures (e.g., failure to stop a pre-potent response when required during the No-go task) or the speed of the inhibitory process (i.e., the stop signal reaction time for the SST) is typically the main outcome measure.

Cognitive inhibition was investigated in ED patients with interference control tasks. These require effortful inhibitory control at a covert cognitive level to suppress the competing automatic response in favour of an alternative response [13]. The most prominent representative of these interference control tasks is the Stroop task. The classical Stroop task elicits a conflict between automatically reading the colour word and naming the incongruent colour of the ink (e.g., the word ‘blue’ is written in green ink colour). Furthermore, modified versions of the Stroop task have been used in ED patients to assess interference control to disease-salient stimuli [20]. Furthermore, the Simon task, which is based on the Simon spatial incompatibility effect (i.e., reaction times are faster and more accurate when the source of stimulation is in the same relative location as the response), also belongs to the group of interference control measures and has been employed in previous studies with bulimic-type ED patients [21], [22].

Other inhibitory control tasks have also been used in previous ED studies to assess primarily response disinhibition. Examples include the Hayling Sentence Completion Test (HSCT), Excluded Letter Fluency task (ELF), and Matching Familiar Figures Test (MFFT) (for further details of these tasks see study concerning [23]).

Previous studies on inhibitory control have reported ambiguous findings with respect to inhibitory control in bulimic-type EDs. Several studies have used the No-go task to assess inhibitory control in bulimic-type EDs [18], [24]–[28]. However, only two of them have reported impaired inhibitory control: one in BN patients (subgroup of BN patients with laxative misuse) [24] and the other in AN-b patients [18]. For the SST, three reports were found, and each included two subtypes of bulimic-type EDs ([9]: in AN-b and BN; [29]: in AN-b and BN; [11]: in BN and BED). However, only one of these studies found significant group differences between BN patients and controls [11], and another reported impairments in AN-b patients [29]. With respect to interference control, the Stroop task has been used most commonly, with inconsistent findings in bulimic-type EDs [28], [30]–[32]. Thus, despite the clinical features and evidence from self- report measures, the current findings from neurocognitive tasks are ambiguous and have not been able to clearly demonstrate impaired inhibitory control in bulimic-type EDs. Furthermore, previous independent studies have frequently used very small sample sizes without reporting effect sizes (ESs). Therefore, it remains unclear whether and to what extent inhibitory control impairment exists in bulimic-type EDs.

Several authors have summarised neurocognitive findings noted in EDs. However, these reviews a) have not conducted meta-analyses to estimate ESs [33], [34]; b) have not included the complete spectrum of bulimic-type EDs [20], [35], [36]; c) have combined currently ill and recovered ED patients [20], [35]; d) have not differentiated between AN-b and AN of the restricting subtype (AN-r) [35], [36]; and e) have only focused on a single type of inhibitory control measure [20], [35].

The aim of the present review with meta-analysis was to summarise the current evidence from multiple neuropsychological studies across the complete spectrum of bulimic-type EDs and across different task categories of inhibitory control to estimate the ES of impaired inhibitory control in patients with binge eating. More specifically, the following questions have been addressed: a) Do bulimic-type EDs (compared to controls) show poorer performance in neurocognitive tasks that address inhibitory control function? b) If so, is this impairment specific to neurocognitive mechanisms of response inhibition (behavioral inhibition) or interference control (cognitive inhibition)? c) Is impaired inhibitory control in bulimic-type EDs greater for disease-salient stimuli (e.g., food) or independent of the type of stimuli? d) Do the effect sizes of impaired inhibitory control differ within the spectrum of bulimic-type ED diagnoses?

## Materials and Methods

### Search strategy and study selection

We searched for published experimental studies on inhibitory control using the following electronic databases: PubMed, PsycINFO, PSYNDEX, and Web of Science. In addition, the reference lists of relevant articles were carefully searched.

The search strategy was based on the following keyword terms: impulsive, impulsivity, inhibitory control, inhibition, disinhibition, attention, executive function, motor control, cognitive control, reward, decision making, neurocognitive, neurocognition, neuropsychology, eating disorder, bulimia nervosa, binge eating disorder, anorexia nervosa, binge/purge, binge eating, purging disorder, and obesity. Searches were limited to human studies, including adults and adolescents of both genders. No date restrictions were applied to the selection of literature, and articles were searched up to March 2013. In addition, we reviewed all studies included in previously published meta-analyses or systematic reviews.

The retrieved titles and abstracts from the literature search were screened for relevance independently by two of the authors (MW, HCF). For every abstract that was identified as potentially relevant by at least one of the two review authors, the full text article was retrieved for evaluation by both reviewers independently. Discrepancies were resolved by discussion. The classification of tasks as inhibitory control measures was based on the information provided in the publications and through discussion among all the authors.

Studies were considered to be acceptable and comparable if they met the following eligibility criteria for inclusion: a) studies had to compare at least one clinical ED group to a healthy control group; b) patients included in the study had to fulfil diagnostic criteria based on the DSM-III, DSM-IV or ICD-10, and results had to be differentiated for current AN of the binge/purge subtype, current BN or current BED; c) studies had to include a detailed description of sociodemographic variables from the healthy control group; d) studies had to include at least one neurocognitive task that investigated inhibitory control; and e) studies had to include sufficient statistical information to allow for the calculations of ESs.

### Data extraction and quality assessment

For data extraction (study characteristics, study results and quality assessment), we used a standardised form developed prior to the search. All discrepancies were rechecked, and disagreements were resolved by discussion with the other authors. Descriptive statistics (means, standard deviations and sample sizes) for the main outcome measures of relevant tasks in bulimic-type ED patients and healthy controls were extracted for the calculation of ESs. When articles did not report means and/or standard deviations, *p* values and sample sizes were used to calculate ESs.

Because no standardized criteria have been established to assess the quality of neuropsychological studies, we developed a priori a standardized checklist of risk of bias which was based on domains of the Newcastle-Ottawa Scale (NOS: www.ohri.ca/programs/clinical_epidemiology/oxford.htm) for evaluating risk of bias in case- control and cohort studies. The NOS consists of the three sub-domains ‘selection of subjects’, ‘comparability of subjects’ and ‘ascertainment of outcome’. We developed for each domain three to four quality items (e.g., inclusion/exclusion criteria, comparability of samples regarding diagnoses, age, educational level, and adequacy of outcome analysis). Each included study was assessed using this ten-item checklist where items were answered either as ‘quality criterion fulfilled (1)’ or as ‘not fulfilled (2)’. Ratings were summed-up to a total score with a maximum value of 10. Quality levels of evidence for each study were defined as high (> = 8), medium (6–8), and low ( = <5). Any discrepancies in quality assessment between the two authors (MW and HCF) were resolved by a third author (MH) who served as an arbiter.

### Quantitative data synthesis

We classified reported outcomes into two categories: a) inhibitory control to general stimuli and b) inhibitory control to disease-salient stimuli (i.e., food/eating and weight/shape stimuli). For articles that included different subtypes of bulimic-type EDs (e.g., AN-b and BN), ESs were calculated separately for each patient group and were treated as separate studies (marked as ‘a’, ‘b’, etc). Separate ESs were calculated for the main outcome of every inhibitory control task in all studies. For studies using more than one neurocognitive measure of inhibitory control [23], [28], a mean ES was computed by averaging ESs across all measures within one study and was included in the calculation of the overall ES.

The ES was calculated as Hedges' *g* (a variation of Cohen's *d* that corrects for biases due to small sample sizes) and reported this value with its 95% confidence interval (CI_95_). The magnitude of Hedges' *g* was interpreted using Cohen's recommendations for small (>0.2), medium (>0.5) and large (>0.8) effect size. A negative ES indicates poorer inhibitory control in patients than in controls. Given the variety of neurocognitive tasks and outcomes, we used the more conservative random-effects model rather than a fixed-effect model to estimate a pooled ES. Heterogeneity among the studies was assessed using the *Q* test. In addition, the *I^2^* statistic values were reported [*I^2^* = (*Q−df*)/*Q*]. As a sample-size independent measure of the inconsistency of ESs across studies, *I^2^* is more powerful with small sample sizes when compared to Cochran's *Q* test. *I^2^*>50% indicates medium heterogeneity, and *I^2^*>75% indicates large heterogeneity [37].

Small study effects as an indication of publication bias were assessed informally by visual inspection of the funnel plot (a plot of effect estimates against its standard error) and using the Egger's test [38].

All analyses were calculated using the software package ‘Comprehensive Meta-Analysis’ (version 2: www.Meta-Analysis.com).

### Moderator analysis and sensitivity analysis

Subgroup analyses of the subtypes of bulimic-type EDs (i.e., AN-b, BN, and BED) and types of inhibitory control measures (e.g., Stroop, No-go, SST) were conducted to identify potential moderators that could explain potential sources of heterogeneity between studies. In addition, sensitivity analyses were conducted to explore the influence of study quality on the pooled overall ES.

## Results

**Figure 1:**
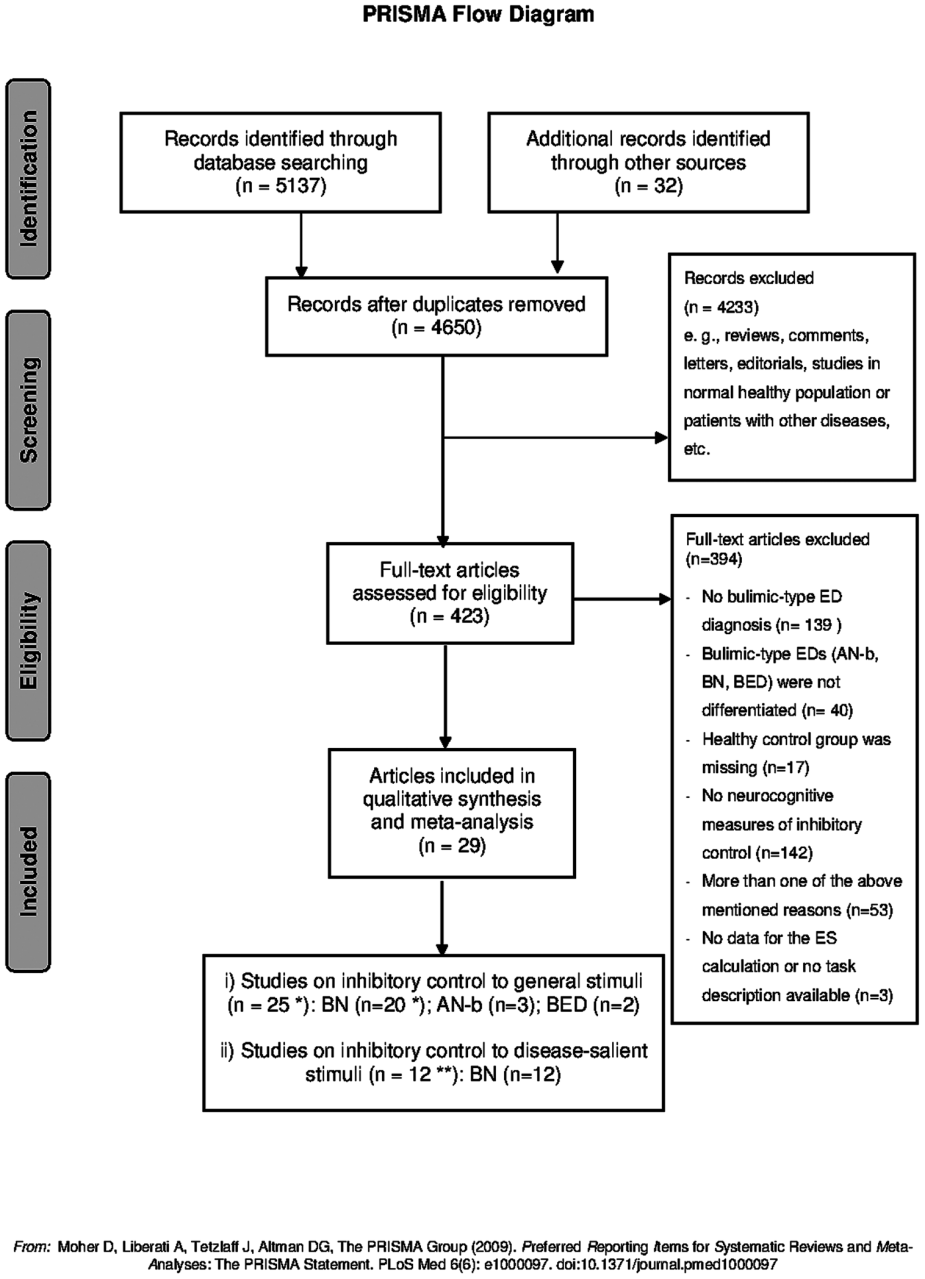
PRISMA Flow Chart. The flow chart highlights the number of articles found at each stage of the search and the final number of studies that were included in the review; *: the number of studies includes one outlier study; **: four articles reported data for inhibitory control to general stimuli and disease-salient stimuli.

The ‘PRISMA statement’ [39] for reporting a systematic review and meta-analyses was followed (see: Checklist S1 for PRISMA items). This PRISMA Flow chart highlights the number of articles found at each stage of the search and the final number of studies that were included in the review (see Figure 1).

Our search resulted in 4650 potentially eligible articles after the exclusion of duplicates. A total of 423 articles appeared to be potentially relevant and were retrieved as full text manuscripts. Of these, 394 articles were excluded because they did not fulfill the inclusion criteria. The reasons for exclusion were as follows: no bulimic-type ED diagnoses (*n* = 139), no differentiation of subtypes of bulimic-type EDs (*n* = 40), no healthy control group (*n* = 17), no neurocognitive tasks or neurocognitive tasks that addressed other cognitive domains (*n* = 142), or more than one of the above mentioned reasons (*n* = 53). In addition, three articles were excluded from the meta-analysis due to a) the lack of appropriate data available for the calculation of ESs [25], [40] or b) insufficient information about neurocognitive task and analysis methods [26]. The qualitative synthesis and meta-analysis were based on the remaining 29 articles that addressed inhibitory control to general and disease-salient stimuli.

### Inhibitory control to general stimuli

#### Study characteristics

Overall, 21 articles, which consisted of 25 studies conducted among bulimic-type ED patients (total of patients: *n* = 583), were included in the meta-analysis. The mean quality score of all included studies was 7.0. The characteristics of the study sample are shown in Table 1.

**Table 1:** Description of studies on general inhibitory control in bulimic-type eating disorders.

Study	Subject	Female (percentage)	Age (years) mean (SD)	BMI (kg/m^2^) mean (SD)	Educational level	Co-morbidity/Treatment/Medication	Task/Outcome variable	Findings	Qualityscore (x/10)
Alvarez-Moya et al. 2009	BN (15)	100%	33.6 (8.8)	26.3 (6)	ns	NO/YES/YES	Stroop/colour-word interference index	ns	7
	HC (15)	100%	35.5 (13.8)	NR					
Ben-Tovim et al. 1989	BN(19)	100%	26.9 (10)	22.61 (4.7)	NR	NR/YES/NR	Stroop/colour-word interference index	ns	6
	HC (38)	100%	22.8 (4.5)	21.31 (2.5)					
Ben-Tovim et al. 1991	BN (27)	NR	26.8 (9.1)*	23.3 (4.6)*	NR	NR/YES/NR	Stroop/colour-word interference index	ns	4
	HC-h (29)	100%	13.6 (1.1)	21.8 (5.7)					
	HC-l (37)	100%	14.0 (1.3)	19.7 (3.5)*					
Black et al. 1997	BN (16)	100%	23.8 (NR)	23 (NR)	NR	NR/NO/NR	Stroop/colour-word time	ns	6
	HC (29)	100%	21.2 (NR)	22.6 (NR)					
Brand et al. 2007	BN (14)	100%	21.9 (3.3)	21.6 (3.8)	ns	NO/NO/NO	Stroop/colour-word interference index	ns	9
	HC (14)	100%	21.6 (2.9)	21.3 (2.3)					
Bruce et al. 2003 (a)	BN (12)	100%	25.6 (5.9)	21.7 (2.3)	NR	YES/YES/NR	No-go/commission errors	BN≠HC*	5
	HC (25)	100%	24.6 (7.1)	22.0 (1.9)					
Bruce et al. 2003 (b)	BN (33)	100%	24.5 (6.7)	22.1 (3.2)	NR	YES/YES/NR	No-go/commission errors	ns	5
	HC (25)	100%	24.6 (7.1)	22.0 (1.9)					
Claes et al. 2006 (a)	AN-b (14)	100%	21.7 (6.8)	NR	NR	NR/YES/NR	Stop signal/SSRT	ns	5
	HC (83)	100%	20.1 (3.1)	NR					
Claes et al. 2006 (b)	BN (22)	100%	22.7 (5.8)	NR	NR	NR/YES/NR	Stop signal/SSRT	ns	5
	HC (83)	100%	20.1 (3.1)	NR					
Cooper et al. 1992	BN (36)	100%	24.3 (6.2)	21.8 (2.2)	NR	NR/YES/NR	Stroop/colour-word interference index	ns	6
	HC (18)	100%	22.1 (3.5)	20.9 (1.5)					
Darcy et al. 2012	BN (23)	100%	16.3(1.2)	109.1(18.2) #	BN<HC*	YES/NR/YES	Stroop/color-word interference index	ns	7
	HC (22)	100%	15.4(1.9)	105.7(12.8) #					
Davidson et al. 2002	BN (17)	100%	25.5 (6.4)	21.2 (3.2)	NR	NR/YES/NR	Stroop/colour-word interference index	ns	6
	HC (18)	100%	24.9 (6.1)	21.1 (2)					
Duchesne et al. 2010	BED (38)	76.3%	33.3 (5.0)	35.9 (2.9)	ns	YES/YES/NO	Stroop/colour-word time	ns	7
	HC (38)	89.5%	35.4 (7.9)	36.6 (3.8)					
Fairburn et al. 1991	BN (24)	100%	21.3 (3.8)	22.6 (3.1)	NR	NR/NR/NR	Stroop/colour-word interference index	BN≠HC*	6
	HC (50)	100%	20.0 (1.1)	20.9 (1.6)					
Galimberti et al. 2012 (a)	AN-b (12)	100%	27.1 (8.9)	15.1 (1.6)*	ns	NO/YES/YES	Stop signal/SSRT	AN-b≠HC*	7
	HC (29)	100%	26.0 (8.4)	19.2 (1.6)					
Galimberti et al. 2012 (b)	BN (16)	100%	25.3 (5.8)	20.4 (3.7)	ns	NO/YES/YES	Stop signal/SSRT	ns	8
	HC (29)	100%	26.0 (8.4)	19.2(1.6)					
Kemps et al. 2010	BN (13)	100%	22.2 (3.9)	23.6 (2.6)	ns	NO/YES/YES	Stroop/colour-word interference index	BN≠HC*	9
	HC (13)	100%	20.8 (3.4)	22.4 (3.4)			HSCT/total score	BN≠HC*	
							ELF/total score	BN≠HC*	
							MFFT/impulsivity score	BN≠HC*	
Marsh et al. 2009	BN (20)	100%	25.7 (7.0)	22.9 (2.3)	ns	NO/YES/YES	SSIT/RT interference	BN≠HC*	8
(outlier)	HC (20)	100%	26.4 (5.7)	22.2 (2.2)					
Marsh et al. 2011	BN (18)	100%	18.4 (2.1)	22.0 (2.0)	ns	YES/NO/YES	SSIT/RT interference	ns	7
	HC (18)	100%	17.3 (2.4)	22.0 (1.9)					
Rosval et al. 2006 (a)	AN-b (16)	100%	25.6 (7.7)	16.7 (1.7)*	NR	NR/YES/NR	No-go/commission errors	AN-b≠HC*	5
	HC (58)	100%	24.3 (6.2)	21.9 (2.2)					
Rosval et al. 2006 (b)	BN (65)	100%	25.0 (6.4)	21.3 (1.9)	NR	NR/YES/NR	No-go/commission errors	ns	6
	HC (58)	100%	24.3 (6.2)	21.9 (2.2)					
Southgate et al. 2008	BN (14)	100%	25.7 (4.9)	21.1 (6.7)	ns	NO/YES/NO	MFFT/impulsivity score	ns	8
	HC (26)	100%	27.3 (11.5)	22.0 (3.4)					
Van den Eynde et al. 2012	BN (40)	100%	28.3 (8.1) *	25.2 (7.2) *	BN≠HC *	NR/YES/YES	Stroop/colour-word interference index	ns	6
	HC (65)	100%	24.0 (2.6)	22.2 (3.3)			No-go/commission errors	ns	
Wu et al. 2013 (a)	BN (16)	93.8%	27.1 (10.2)	22.2 (2.9)	ns	YES/YES/YES	Stop signal/SSRT	BN≠HC*	8
	HC (25)	96.0%	26.3 (5.4)	22.1(2.0)					
Wu et al. 2013 (b)	BED (44)	90.9%	40.1 (11.6)	34.0 (5.0)	BED≠HC *	YES/YES/YES	Stop signal/SSRT	ns	8
	HC (39)	97.4%	39.8 (11.3)	35.1 (5.1)					

BN: bulimia nervosa; BED: binge eating disorder; AN-b: anorexia nervosa from the binge/purge subtype; HC: healthy controls; BMI: body mass index;

^#^ : ideal body weight (percentage);

ns: no significant difference between patients and healthy controls;

significant group difference; NR: not reported; SSRT: stop signal reaction time; MFFT: Matching Familiar Figure Test; HSCT: Hayling Sentence Completion Test; ELF: Excluded Letter Fluency test; SSIT: Simon Spatial Incompatibility Task; RT: reaction time.

Twenty studies were conducted in BN patients (total of patients: *n* = 459, sample sizes ranging from 12 to 65), three studies in AN-b patients (total of patients: *n* = 42, sample sizes ranging from 12 to 16) and two studies in BED patients (patients: *n* = 38, and 44). Most participants in the included studies were female, with the exception of two articles that had a few male participants [11], [32] (see Table 1). The mean age of the entire sample was 26.4 years and ranged from 16.3 to 40.1 years (two articles [22], [41] included adolescent BN patients). The mean ages for the ED subtypes were 24.7, 24.6, and 36.9 years for the AN-b, BN, and BED subgroups, respectively. The mean BMI of the entire sample was 24.0 kg/m^2^, ranging from 15.1 to 35.9 kg/m^2^. The mean BMI for the ED subtypes were 17.9, 22.5, and 34.9 kg/m^2^ for the AN-b, BN, and BED subgroups, respectively. Approximately half of the studies provided information about educational levels or IQ scores (see Table 1). Two studies did not mention whether the patients were currently under treatment [42], [43]. Eleven studies did not provide data concerning comorbidity, while seven studies excluded participants with comorbid diagnoses, e.g., obsessive-compulsive disorder, substance abuse or dependence, psychotic disorder, bipolar disorder, etc. (see Table 1). Three studies reported the exclusive inclusion of medication-free participants [31], [32], [44], however, half of the studies did not mention or specify the usage of medication (see Table 1).

Of note, of the individual studies, 17 of the 25 (sample sizes of patients range from 14 to 65) failed to identify significant impairments in general inhibitory control in bulimic-type ED patients (see Table 1).

#### Small study effects and overall effect size

**Figure 2:**
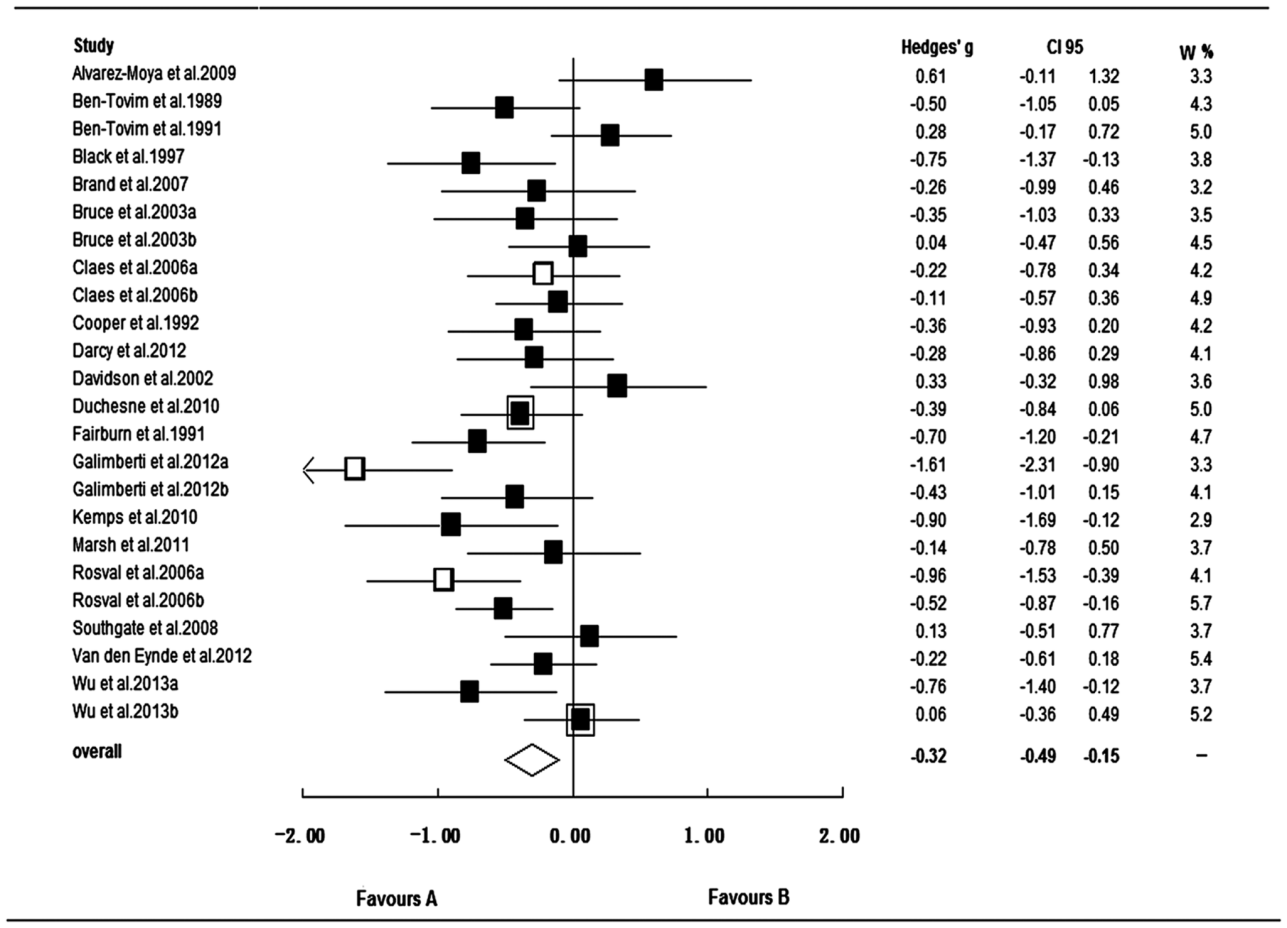
Forest plot for studies on inhibitory control to general stimuli in bulimic-type eating disorders. **▪**: bulimia nervosa; **▪ with frame**: binge eating disorder; **□** anorexia nervosa from the binge/purge subtype; CI95: 95% confidence interval; W %: relative weight (percentage); Favours A/B: lower/higher inhibitory control in bulimic-type EDs than in controls.

Visual inspection of the funnel plot suggested no small study effects, which was confirmed by a non-significant result on the Egger's test (*p* = 0.457). However, visual inspection of the funnel plot suggested a potential outlier (see Figure S1). The ES of the study [21] (20 BN patients compared to 20 controls) was −2.9 (*SE* = 0.45, CI_95_ = [−3.77; −2.02]). The pooled overall ES including the study was −0.40 (*SE* = 0.11, CI_95_ = [−0.61; −0.19], *p*<0.001), *Q* (24) = 86.0, *p*<0.001, *I^2^* = 72.1%. After excluding the outlier study, the overall ES of the remaining studies on general inhibitory control was −0.32 (*SE* = 0.09, CI_95_ = [−0.49; −0.15], *p* = 0.001), and the heterogeneity between studies remained significant, *Q* (23) = 53.1, *p*<0.001 (see Figure 2); however, the ESs across studies decreased to medium inconsistency (*I^2^* = 56.7%). Thus, we excluded the outlier study from the following analysis.

#### Moderator analysis and sensitivity analysis

Subgroup analyses for the subtypes of bulimic-type EDs revealed significant negative effects in AN-b and BN, indicating impaired inhibitory control in this patient subpopulation. The ES for AN-b (Hedges' *g* = −0.91) was higher than that for BN (Hedges' *g* = −0.26), although this difference did not reach statistical significance. In addition, the ESs across the three AN-b studies was inconsistent to a larger extent (*I^2^* = 78.7%) than that across the 19 BN studies (*I^2^* = 42.7%). The pooled ES for two BED studies was non-significant (Hedges' *g* = −0.16, p = 0.485) (see Table 2).

**Table 2:** Moderator analysis of studies on general inhibitory control in bulimic-type eating disorders.

	Number of studies	Number of patients	Effect size				Heterogeneity		Small study effects
			Hedges' *g*	CI_95_	*SE*	*p* value			*p* value (Egger's test)
**subtype of eating disorders**									
BN	19	439	−0.26	−0.43, −0.09	0.09	0.003	Q (18) = 31.4, p = 0.026	I^2^ = 42.7%	0.878
AN-b	3	42	−0.91	−1.67, −0.14	0.39	0.020	Q (2) = 9.38, p = 0.009	I^2^ = 78.7%	0.375
BED	2	82	−0.16	−0.60, +0.28	0.22	0.485	Q (1) = 2.0, p = 0.156	I^2^ = 50.3%	—
**tasks**									
Stroop	12	282	−0.25	−0.47, −0.02	0.12	0.035	Q (11) = 22.0, p = 0.025	I^2^ = 49.9%	0.957
SST	6	124	−0.46	−0.90, −0.03	0.22	0.036	Q (5) = 18.6, p = 0.002	I^2^ = 73.2%	0.009
No-go	5	165	−0.39	−0.69,−0.09	0.15	0.010	Q (4) = 7.72, p = 0.102	I^2^ = 48.2%	0.905
MFFT	2	27	−0.35	−1.34, +0.64	0.50	0.488	Q (1) = 3.86, p = 0.05	I^2^ = 74.1%	—
HSCT	1	13	−1.09	−1.89, −0.29	0.41	0.008	—	—	—
SSIT	1	18	−0.14	−0.78, +0.50	0.33	0.662	—	—	—
ELF	1	13	−1.02	−1.81, −0.23	0.41	0.012	—	—	—

CI_95_: 95% confidence interval, SE: standard error; BN: bulimia nervosa; BED: binge eating disorder; AN-b: anorexia nervosa from the binge/purge subtype; SST: Stop Signal Task; MFFT: Matching Familiar Figure Test; HSCT: Hayling Sentence Completion Test; SSIT: Simon Spatial Incompatibility Task; ELF: Excluded Letter Fluency test.

Various tasks were used to investigate general inhibitory control in the retrieved studies, with the Stroop, SST, and No-go tasks being the most commonly used. The ESs for the individual task categories are presented in Table 2. Differences in ESs across task categories did not reach significance (*p* = 0.276). The pooled ES for studies of cognitive interference control (i.e., 12 studies using the Stroop task and one study using the Simon task) was −0.24 (*SE* = 0.11, CI_95_ = [−0.45; −0.03], *p* = 0.027). The pooled ES for studies on response inhibition (i.e., six studies using the SST and five studies using the No-go task) was −0.42 (*SE* = 0.13, CI_95_ = [−0.67; −0.17], *p* = 0.001). With respect to disinhibition, only two studies were available. In one of these studies, the MFFT was used in a sample of 14 BN patients and showed a non-significant ES (Hedges' *g* = 0.13, *SE* = 0.33, CI_95_ = [−0.51, 0.77], *p* = 0.692) [44], while in the other study, the MFFT, HSCT, and ELT were used in 13 BN patients and revealed ESs with Hedges' *g* of −0.88 for MFFT, −1.09 for HSCT, −1.02 for ELT (all *p* values <0.03) [23]. There were not enough studies on disinhibition to calculate a pooled ES.

The sensitivity analysis, which included only studies of medium to high quality (18 of 24 studies), yielded a comparable overall ES (Hedges' *g* = −0.36, CI_95_ = [−0.56; −0.16]), showing that the study quality did not influence the pooled ES in a significant way.

### Inhibitory control to disease-salient stimuli

#### Study characteristics

A total of 12 studies in bulimic-type ED patients (total *n* = 218, sample size ranging from 10 to 30) using neurocognitive tasks on inhibitory control to disease-salient stimuli (food/eating, shape/weight) were included in the meta-analysis. The mean quality score of all included studies was 6.2. The characteristics of the study sample are shown in Table 3.

**Table 3:** Description of studies on disease-related inhibitory control in bulimic-type eating disorders.

Study	Subject	Female (percentage)	Age (years) mean (SD)	BMI (kg/m^2^) mean (SD)	Educational level	Co-morbidity/Treatment/Medication	Task/Outcome variable	Findings	Quality score (x/10)
Ben-Tovim et al. 1989	BN(19)	100%	26.9 (10)	22.6 (4.7)	NR	NR/YES/NR	Stroop/interference index (food)	BN≠HC *	6
	HC (38)	100%	22.8 (4.5)	21.3 (2.5)			Stroop/interference index (shape)	BN≠HC *	
Ben-Tovim et al. 1991	BN (27)	NR	26.8 (9.1)*	23.3 (4.6)*	NR	NR/YES/NR	Stroop/interference index (food)	BN≠HC *	4
	HC-h (29)	100%	13.6 (1.1)	21.8 (5.7)			Stroop/interference index (shape)	BN≠HC *	
	HC-l (37)	100%	14.0 (1.3)	19.7 (3.5)*					
Black et al.1997	BN (16)	100%	23.8 (NR)	23 (NR)	NR	NR/NO/NR	Stroop/interference index (food)	ns	6
	HC (29)	100%	21.2 (NR)	22.6 (NR)			Stroop/interference index (shape)	ns	
Cooper et al. 1997	BN (12)	100%	NR	NR	NR	NR/YES/NR	Stroop/interference index (eating)	BN≠HC *	6
	HC (18)	100%	NR	NR			Stroop/interference index (weight/shape)	BN≠HC *	
Davidson et al. 2002	BN (17)	100%	25.5 (6.4)	21.2 (3.2)	NR	NR/YES/NR	Stroop/interference index (food)	BN≠HC *	6
	HC (18)	100%	24.9 (6.1)	21.1 (2.0)			Stroop/interference index (shape)	BN≠HC *	
Flynn et al. 1999	BN (15)	100%	27.1 (7.0)*	98.2 (7.1)#	ns	NR/YES/NR	Stroop/interference index (food)	ns	6
	HC (13)	100%	22.3 (4.7)	95.2 (7.2)#			Stroop/interference index (body)	BN≠HC *	
Jones-Chesters et al. 1998	BN (16)	100%	25.6 (7.7)	23.8 (3.0)	ns	NR/YES/NR	Stroop/RT (food/eating)	BN≠HC *	7
	HC (16)	100%	26.6 (7.5)	22.1 (2.8)			Stroop/RT (weight/shape)	BN≠HC *	
Lokken et al. 2006	BN (30)	100%	19.1 (1.4)	22.1 (4.3)	ns	NR/NR/NR	Stroop/interference index (eating)	BN≠HC *	7
	HC (30)	100%	19.5 (1.0)	22.2 (2.5)			Stroop/interference index (weight/shape)	BN≠HC *	
Lovell et al. 1997	BN (24)	100%	26.9 (11.1)	21.8 (3.5)	ns	NR/YES/NR	Stroop/RT (food)	ns	6
	HC (33)	100%	24.7 (8.1)	22.9 (3.6)			Stroop/RT (shape)	BN≠HC *	
Mobbs et al. 2008	BN (18)	100%	25.1 (3.9)	20.4 (2.6)	ns	YES/YES/NR	No-go/decision bias (food)	BN≠HC *	8
	HC (18)	100%	24.3 (3.4)	21.0 (1.6)			No-go/decision bias (body)	ns	
Perpiná et al. 1993	BN (14)	100%	26.4 (4.9)	26.6 (8.3)	ns	NR/YES/NR	Stroop/RT (food)	ns	7
	HC (32)	100%	26.9 (6.0)	23.4 (3.5)			Stroop/RT (body)	BN≠HC *	
Perpiná et al. 1998	BN (10)	100%	27.8 (8.2)	21.1 (2.2)	NR	NR/YES/NR	Stroop/interference index (food)	NR	5
	HC (18)	100%	29.3 (9.7)	21.1 (2.7)			Stroop/interference index (shape)	NR	

BN: bulimia nervosa; BED: binge eating disorder; AN-b: anorexia nervosa of the binge/purge subtype; HC: healthy controls; BMI: body mass index;

^#^ : ideal body weight (percentage);

ns: no significant difference between patients and healthy controls;

significant group difference;

NR: not reported; RT: reaction time.

All the studies were conducted in BN patients. With the exception of one study, which did not report the gender of participants [45], samples tended to be exclusively female. The mean age of patients was 25.5 years, with a range from 19.1 to 27.8 years, and the mean BMI was 22.3 kg/m^2^, with a range from 20.4 to 26.6 kg/m^2^. Approximately half of the studies provided information about educational levels or IQ scores. Only one study included data on co-morbid diagnoses [46], and none of the studies provided information about the usage of psychotropic medication.

Four out of the 12 studies (sample sizes of patients ranging from 14 to 24) failed to identify significant impairments in inhibitory control to food/eating stimuli in BN patients, and two out of the 12 studies (sample sizes of patients were 16 and 18) failed to identify significant impairments in inhibitory control to body/shape stimuli in BN patients (see Table 3).

#### Small study effects and overall effect size

Visual inspection of funnel plots suggested no small study effects for inhibitory control to food/eating or to shape/weight, which were confirmed by the results of the Egger's test (*p* = 0.933; *p* = 0.532).

**Figure 3:**
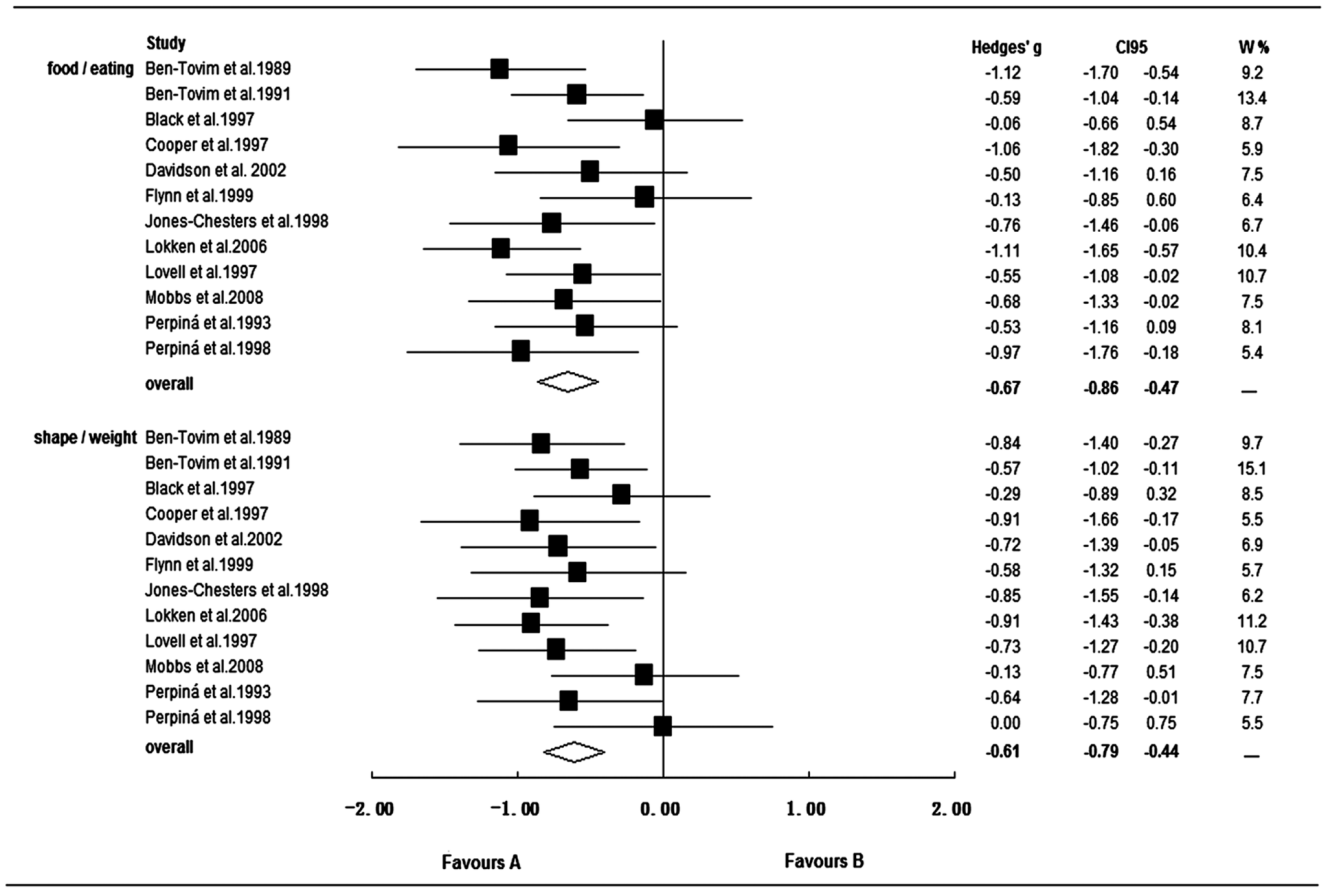
Forest plot for studies on inhibitory control to disease-salient stimuli in bulimia nervosa patients. **▪**: bulimia nervosa; CI95: 95% confidence interval; W %: relative weight (percentage); food/eating: study used food/eating related stimuli; shape/weight: study used shape/weight related stimuli; Favours A/B: lower/higher inhibitory control in bulimic-type EDs than in controls.

The overall ES for studies on inhibitory control to food/eating stimuli was medium (Hedges' *g* = −0.67, *SE* = 0.10, CI_95_ = [−0.86; −0.47], *p*<0.001). No significant evidence of heterogeneity was found, *Q* (11) = 13.4, *p* = 0.266, *I^2^* = 18.1%. Studies on inhibitory control to shape/weight stimuli in BN patients also showed impairments with a medium ES (Hedges' *g* = −0.61, *SE* = 0.09, CI_95_ = [−0.79; −0.44], *p*<0.001), with no indication for heterogeneity, *Q* (11) = 9.1, *p* = 0.616, *I^2^* = 0. There was no significant difference between ESs for food/eating and shape/weight (see Figure 3). However, the overall ES for inhibitory control to disease-salient stimuli was significantly larger than that for general inhibitory control (*p* = 0.014).

#### Moderator analysis and sensitivity analysis

As Table 4 shows, all studies were run in BN patients, therefore, no comparison between subtypes of bulimic-type EDs was possible.

**Table 4:** Moderator analysis of studies on inhibitory control to disease-salient stimuli in BN patients.

	Number of studies	Number of patients	Effect size				Heterogeneity		Small study effects
			Hedges' *g*	CI_95_	*SE*	*p* value			*p* value (Egger's test)
a) **Food/eating**									
BN	12	218	−0.67	−0.86, −0.47	0.10	<0.001	Q (11) = 13.4, p = 0.266	I^2^ = 18.1%	0.933
**Tasks**									
Stroop	11	200	−0.67	−0.88, −0.45	0.11	<0.001	Q (10) = 13.4, p = 0.201	I^2^ = 25.5%	0.938
No-go	1	18	−0.68	−1.34, −0.02	0.34	0.042	—	—	—
b) **Shape/weight**									
BN	12	218	−0.61	−0.79, −0.44	0.09	<0.001	Q (11) = 9.1, p = 0.616	I^2^ = 0	0.532
**Tasks**									
Stroop	11	200	−0.65	−0.84, −0.47	0.09	<0.001	Q (10) = 6.65, p = 0.758	I^2^ = 0	0.628
No-go	1	18	−0.13	−0.77, +0.51	0.33	0.699	—	—	—

CI_95_: 95% confidence interval, SE: standard error; BN: bulimia nervosa.

The Stroop task was used most frequently (11 out of the 12 studies), showing significant negative ESs for cognitive interference control to food/eating and shape/weight stimuli in BN patients (Hedges' *g* = −0.67, −0.65, respectively). There was only one study in which an alternative task (i.e., No-go task) was used to measure response inhibition [46]. The ES for the No-go study showed a significant effect for impaired inhibitory control to food/eating stimuli (Hedges' *g* = −0.68, *p* = 0.042) but not to shape/weight stimuli (*p* = 0.699) in BN patients (see Table 4).

Sensitivity analyses including only studies of medium to high quality (10 of the 12 studies) yielded comparable overall ESs (food/eating: Hedges' *g* = −0.66, CI_95_ = [−0.90; −0.41]; shape/weight: Hedges' *g* = −0.67, CI_95_ = [−0.86; −0.47]), demonstrating that the study quality did not influence the pooled ESs in a significant manner.

## Discussion

The present systematic review and meta-analysis is the first to quantitatively synthesise the neuropsychological findings of inhibitory control in bulimic-type EDs, which consist of AN-b, BN and BED. Publications were searched up to March 2013, and 24 studies assessing inhibitory control to general stimuli and 12 studies focusing on inhibitory control to disease- salient stimuli were included in the meta-analysis.

The main finding of the meta-analysis is that bulimic-type ED patients have impaired general inhibitory control with a small ES when compared to healthy controls. The two main task categories of response inhibition (i.e., No-go task, SST) and effortful cognitive interference control (i.e., Stroop task, Simon task) showed a similar level of impairment. Notably, there was a significantly greater impairment (medium ES) to disease-salient stimuli than general stimuli, indicating that disease-salient stimuli worsen the general impairment of inhibitory control in BN patients. The comparison of inhibitory control to general and disease-salient stimuli was restricted to BN patients due to a lack of studies in AN-b and BED patients. Furthermore, the findings were largely based on cognitive interference control tasks (i.e., the Stroop task). Further research is needed to investigate whether greater impairment to disease-salient stimuli is also found in AN-b and BED and whether greater impairment is task- category independent and may also be found in response inhibition tasks. Differentiating the spectrum of bulimic-type EDs using diagnostic categories, preliminary findings indicate a large ES of AN-b, a small ES for BN, and a non-significant ES for BED with respect to impaired inhibitory control to general stimuli. However, these findings should be treated with caution, as most of the studies were conducted with BN populations, while only three studies were conducted in AN-b patients, and only two studies in BED patients were available.

As an extension of findings from previous studies, the present meta-analysis is the first to show significantly greater impairments of inhibitory control to disease-salient stimuli compared to general stimuli in BN patients. This finding indicates that attentional bias and approach motivation to disease-salient stimuli in BN patients may potentiate the generally impaired inhibitory control in this population, thus facilitating binge eating (releasing feeding behaviours from regulatory control). In comparison to previous meta-analyses, the enlarged meta-analysis of the present study showed a slightly smaller ES for impaired general inhibitory control in BN when compared to previous reviews [20], [36]. In contrast, slightly higher effects were found in BN patients to disease-salient stimuli in the present meta-analysis when compared to previous reports [20], [35]. However, the inclusion of recovered patients in previous meta-analyses [20], [35] may have also contributed to the mentioned differences in ESs.

Furthermore, with respect to a dimensional approach, the present findings support a decrease in inhibitory control from BED over BN to AN-b patients. This finding has clinical implications for the ED psychopathology, as it suggests that in BED patients, decreased inhibitory control seems to be of less importance than for AN-b and BN patients. These differences in inhibitory control may be relevant to behavioural distinctions for binge eating between BN and BED patients [11]. However, further research is needed to support the preliminary findings in BED patients.

The present review has progressed findings from previous reviews in a number of ways. First, this review has included a larger sample of bulimic-type ED patients (a total of 563 patients), thus covering the broad spectrum of bulimic-type EDs (i.e., AN-b, BN, and BED). Second, the methodological quality of this review was systematically assessed and included in sensitivity analyses, showing that there was no evidence that studies of lower methodological quality confounded the estimated ESs. In addition, in comparison to previous meta-analyses that focused on one specific inhibitory control task (i.e., Stroop task) [20], [35], we included different inhibitory control tasks to calculate a more comprehensive ES of impaired inhibitory control in bulimic-type EDs. This methodological approach was justified, as heterogeneity (*I* ^2^ value) was acceptable in the face of rather different tasks and patient populations. The stability of ESs over different inhibitory control tasks suggests a common underlying mechanism that seems independent from the specific task. Of note, for general inhibitory control, more than half of clinical studies failed to identify significant impairments in bulimic-type ED patients. In contrast, the meta-analysis with higher statistical power uncovered a small but significant impaired inhibitory control in bulimic-type ED patients.

It was not until recently that researchers began to investigate the underlying neural circuits of impaired inhibitory control in bulimic-type EDs [21], [22], [47], [48]. These neuroimaging studies suggest altered brain activation within frontostriatal circuits during the execution of inhibitory control tasks. More specifically, BN patients failed to activate frontostriatal circuits to the same degree as healthy comparison subjects during the execution of a cognitive inhibitory control task (Simon task: [21], [22]; No-go task: [47]). Further evidence for dysfunctional lateral prefrontal cortex activity comes from a study using transcranial magnetic stimulation [49]. These altered brain activations within frontostriatal circuits may contribute to deficits in inhibitory control at a neurocognitive level in bulimic- type EDs. Given the findings of the present meta-analysis, determining the neural mechanisms that contribute to deterioration in inhibitory control to disease-salient stimuli in BN patients may be a promising avenue for future neuroimaging research.

### Limitations and implications for future work

First, the fact that we excluded studies that did not provide data for ES calculations [25], [40] and the lack of a direct search for unpublished data and grey literature may have resulted in a potential bias, which might have influenced the stability of pooled overall ES for inhibitory control. However, in the current review, the funnel plot and the Egger's test did not show small study effects, which is an indicator of the publication bias. Second, as common to all meta-analyses, findings from the current meta-analyses are to some extent influenced by the quality of primary studies, although the sensitivity analyses demonstrated that the quality levels of the included studies did not significantly affect the overall ES. We developed a priori a standardized checklist of risk of bias which was based on the domains of the Newcastle-Ottawa Scale, as no standardized criteria have been developed to assess the quality of neuropsychological studies. Third, for some studies, the data for the main outcome measures were not available (e.g., interference index for Stroop task), and the secondary outcome measure had to be used (e.g., score from colour-word conflict trial). This may have increased the heterogeneity across studies. However, the statistical testing for heterogeneity across studies showed acceptable inconsistencies (after excluding one potential outlier study). Fourth, our findings of impaired inhibitory control are valid for bulimic-type ED patients in the acute phase. We did not include patients who had recovered from bulimic-type EDs and did not consider whether patients were in treatment during investigation. Further research in ED patients after recovery or rather longitudinal studies are needed to differentiate whether inhibitory impairments are a state or rather a trait or a ‘scar’ after recovery of bulimic-type eating disorders. Fifth, we have not considered emotional interference on inhibitory control performance that may be of relevance in ED patients. However, at present the number of studies is too small to calculate a quantitative meta-analysis across ED diagnoses. Finally, the estimated ESs for AN-b and BED patients should be treated with caution given the limited sample sizes.

These findings have significant clinical implications, as dimensional measures of neurocognitive functioning related to inhibitory control may help to select specific psychological (e.g., cognitive remediation), as well as pharmacological interventions (e.g. selective serotonin reuptake inhibitors) that are designed to target basic neurobiological processes involved in inhibitory control functions. A better understanding of the neurocognitive profiles of bulimic-type EDs may help to provide a more fine-grained diagnostic classification, and refine existing treatment approaches as well as inform the development of new interventions.

Given the impaired inhibitory control noted in the studies, especially to disease-salient stimuli, these findings support the use of impulse control techniques in the current treatment regimens. In addition, cognitive training to address inhibitory control (i.e., cognitive remediation therapy) may improve treatment outcome and may represent a promising treatment modality for future research [50]. With respect to experimental research, future studies should consider more complex paradigms that would allow for the further exploration of the interactions between inhibitory control and other cognitive domains (e.g., set-shifting, decision-making) as well as emotional interference. Given the high comorbidity of depression and anxiety disorders in EDs, future studies should consider to a greater extent the impact of mood states on the performance in inhibitory control tasks or to use paradigms that systematically differentiate between with and without emotional interference.

## Conclusions

The primary finding of the present review is that bulimic-type ED patients show an impairment in inhibitory control to general stimuli with a small ES. This rather low general impairment in BN patients was enlarged by disease-salient stimuli, suggesting a greater impairment of inhibitory control specifically to disorder related stimuli, which may underlie recurrent episodes of binge eating. For AN-b and BED patients, further clinical studies are needed to validate the preliminary findings.

## Supporting Information

Checklist S1 **PRIMSA Checklist.**
(DOC)

Figure S1 **Funnel plot for all studies on general inhibitory control in bulimic-type eating disorders.** The arrow indicates one outlier study.
(TIF)
