# Supplementary figures and images for: Inhibitory Control in Bulimic-Type Eating Disorders: A Systematic Review and Meta-Analysis
Source: PLoS One. 2013 Dec 31;8(12):e83412. doi: 10.1371/journal.pone.0083412 (PMC3877018; doi:10.1371/journal.pone.0083412)

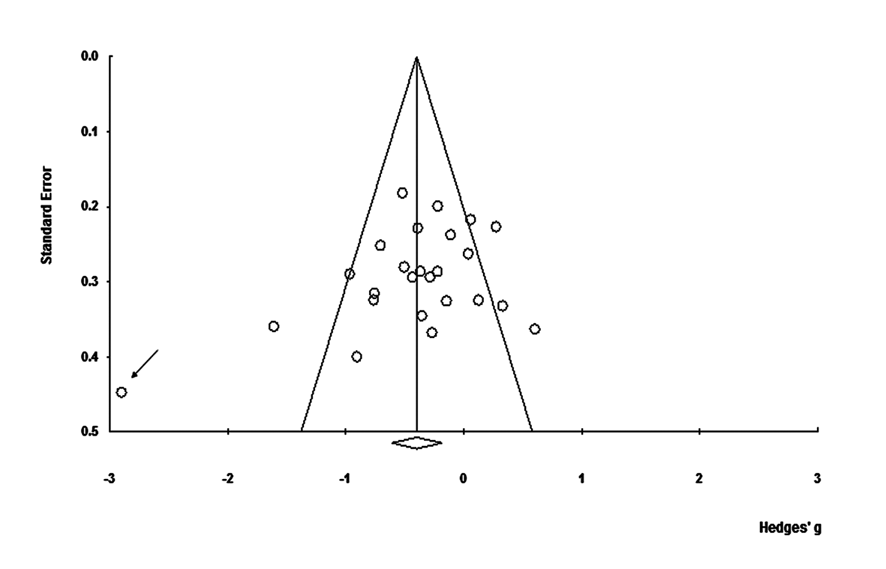

Supplement: Figure S1 — Funnel plot for all studies on general inhibitory control in bulimic-type eating disorders. The arrow indicates one outlier study. (TIF) [file pone.0083412.s002.tif]
